# Redefine oral health: a call for inclusivity in the concept of oral health

**DOI:** 10.3389/froh.2025.1543770

**Published:** 2025-05-07

**Authors:** Moréniké Oluwátóyìn Foláyan, Nicaise Ndembi, Olunike Rebecca Abodunrin, Bridget Haire

**Affiliations:** ^1^ The Africa Oral Health Network (AFRONE), Alexandria University, Alexandria, Egypt; ^2^ Department of Child Dental Health, Obafemi Awolowo University, Ile Ife, Nigeria; ^3^ Director General's Office, International Vaccine Institute (IVI), Africa Regional Office, Kigali, Rwanda; ^4^ Division of Epidemiology and Prevention, Institute of Human Virology, University of Maryland School of Medicine, Baltimore, MD, United States; ^5^ Department of Epidemiology and Biostatistics, Nanjing Medical University, Nanjing, Jiangsu, China; ^6^ School of Population Health and Kirby Institute, University of New South Wales, Sydney, NSW, Australia

**Keywords:** sexual behavior, psychosocial factors, self-concept, health equity, health policy, health disparities, sexual and gender minorities, quality of life

## Abstract

Current, globally accepted definitions of oral health emphasize pain-free functionality, expressive capacity, and specific psychosocial dimensions that contribute to self-confidence, well-being, and societal participation. However, these definitions inadvertently exclude diverse lived experiences by framing oral health solely through a lens of “normal” functionality and absence of discomfort, failing to consider the ways in which oral health can be experienced uniquely by different individuals and communities. The narrow focus on “pain-free” oral health excludes valid aspects of sexual expression, which may involve consensual oral activities that some find pleasurable even if associated with discomfort. This manuscript examines the limitations of the WHO and FDI definitions of oral health, critiques their exclusion of minority perspectives, and advocates for a more inclusive, holistic approach. Such an approach recognizes the complex and varied ways oral health intersects with identity, intimacy, and societal norms. Normalizing discussions on oral sexual health are essential to advancing a comprehensive understanding of well-being and reducing stigma around sexual minority experiences. Expanding oral health definitions to accommodate broader conceptions of intimacy and pleasure can enhance public health policy, clinical practice, and education, fostering a comprehensive understanding of well-being that reduces stigma around sexual minority experiences and further marginalization of sexual minorities in accessing inclusive care.

## Introduction

The concept of oral health as pain-free functionality is enshrined in two prominent, globally accepted definitions of oral health. These two definitions differ somewhat in their emphasis: the FDI World Dental Federation's definition centers on the absence of pain, disease, and discomfort (1) and aims to ensure functional and expressive well-being. The definition by the World Health Organization describes oral health as a state enabling essential functions—eating, breathing, speaking—and encompassing dimensions such as self-confidence, societal participation, and freedom from discomfort or embarrassment (2). These concepts of oral health align with traditional views of health as freedom from suffering, allowing individuals to eat, speak, smile, and communicate with ease.

Together, the definitions emphasize the biopsychosocial model of health, including (3) the connection between oral health and general well-being, and this perspective supports vital aspects of well-being (4). The model is celebrated because it offers a holistic approach to understanding health and illness, integrating biological, psychological, and social dimensions into the understanding of health (5). This broader perspective acknowledges the significant role that mental health, social environments, and personal behaviors play in influencing health outcomes (6). It contrasts with the traditional biomedical model, which primarily focuses on the biological aspects of health (7). The biopsychosocial model promotes personalized care by encouraging healthcare providers to create treatment plans that address not only physical symptoms but also psychological issues, such as stress, and social factors, such as family dynamics or socioeconomic conditions (8).

Further, the biopsychosocial model emphasizes the importance of prevention. Recognizing the interconnectedness of mental and social well-being, it encourages patients to actively manage their health, adopt behavioral changes, and develop coping strategies (9). This focus on prevention extends to promoting better doctor-patient relationships, where understanding a patient's psychological and social contexts can enhance communication, trust, and the overall effectiveness of care (10). The model is particularly beneficial in managing chronic illnesses, where psychological stress and social conditions often exacerbate physical symptoms (11).

However, the biopsychosocial model of oral health has its limitations. Its broad and integrated approach can be challenging to implement in busy clinical settings due to time constraints, limited resources, and a lack of training in non-medical areas like psychology and social work (9). In addition, psychological and social factors can be subjective and difficult to measure, leading to inconsistencies in care. While there is substantial evidence supporting the role of biological factors in health, there is less research on the effectiveness of interventions targeting psychological and social aspects, making it harder to standardize treatment protocols (12). The inclusivity of the model can also lead to overgeneralization, as it may be difficult to determine the relative contribution of each factor to a patient's health, potentially complicating accurate diagnoses (13). Furthermore, the effective application of the model requires a wide range of healthcare professionals and resources, which may not be readily available, particularly in low-resource settings, including many African countries (8).

Moreover, as a key determinant of overall health and well-being, oral health encompasses various social and cultural dimensions. For some, oral health experiences involve nuanced dynamics of pleasure and pain, which are valid elements of their lived realities (14). The narrow view of oral health where wellness and well-being are defined as the absence of pain and discomfort overlooks the experiences of a minority of people, for whom pain and discomfort may not preclude wellness and well-being. In effect, discomfort and pain are not universally perceived as negative, and their absence does not always reflect or contribute to wellness and well-being.

### The current definition and its limitations

The current concept of oral health is framed in a somewhat binary way—defined by the presence or absence of disease or discomfort. However, this narrow understanding of health as simply the absence of disease fails to capture the full complexity of oral wellness and well-being. Discomfort and pain, often seen as negative indicators of oral health, are not universally perceived in the same way across cultures or even within individuals. The absence of discomfort or pain does not necessarily correlate with the complete absence of underlying health issues, nor does it always imply that an individual is in a state of well-being (15, 16). For example, individuals who have oral diseases, such as gingivitis or incipient cavities that are not causing pain or discomfort, may still experience lasting effects from the diseases on their well-being.

In addition, cultural perceptions of pain and discomfort can vary significantly (17). In some cultures, pain might be viewed as a normal part of life or a minor inconvenience, while in others, it might be considered a more significant marker of poor health. This subjectivity means that the absence of pain or discomfort is not a universally reliable measure of oral health, and it is possible for someone to feel physically well while experiencing significant psychological or social distress related to their oral health (18). For instance, an individual with cosmetic dental issues that do not cause pain may still feel socially isolated or experience a reduction in quality of life due to low self-esteem or social stigma.

Of concern also is the failure of the framing of oral health to take account of the experiences of those who associate oral experiences, such as certain forms of intimacy, with positive sensations, regardless of discomfort. Individuals may have positive experiences associated with consensual oral activities that may not always be pain-free but are nevertheless consensually pleasurable.

This restrictive understanding may contribute to an environment where oral health professionals lack the language or frameworks to engage with patients on diverse oral health experiences, perpetuating silence and stigma around topics such as oral sexual health. While a pain-free life may be ideal, for some, certain types of controlled pain or discomfort may be integral and acceptable aspects of life that, when related to sexual satisfaction, may be closely linked to well-being. Consequently, framing oral health solely through an absence of pain may inadvertently negate the diversity of intimate practices. This oversight becomes particularly critical in the context of public health, where definitions and frameworks influence clinical practices, health education, and policies. A limited understanding of oral health may encourage judgment or silence in clinical settings, limiting opportunities to discuss, normalize, and support diverse oral health behaviors.

### Pain, pleasure, and oral health: reframing definitions

Oral health can encompass varied expressions of identity and intimacy that may involve consensual discomfort or pain, challenging conventional definitions of health. For some, experiences like oral sexual health are integral to their well-being and identity (19). A more inclusive oral health framework that recognizes these realities could enable health policies that validate diverse expressions of intimacy and selfhood. By normalizing discussions around oral sexual health, health systems can better support individuals’ psychosocial well-being, contributing to a holistic understanding of health that respects personal experiences and preferences.

Oral sex is a sexual activity with genital stimulation using the mouth, tongue, teeth, or throat, including oral-vaginal/clitoral contact (cunnilingus), oral-penile contact (fellatio), and oral-anal contact (anilingus). It can be practiced by sex partners of all sexualities (20). Performing oral sex may cause discomfort for some, but this does not necessarily reduce sexual satisfaction, and there is evidence that oral sex enhances well-being and sex life, especially in older adults (21, 22).

### Prevalence of oral sex

When definitions of oral health are framed narrowly or without consideration of the full range of oral behaviors, such as oral sex, they can overlook key aspects of oral health and wellness that affect a large proportion of the population. We conducted a rapid review of the literature to identify publications on the prevalence of oral sex. The search was conducted in PubMed and the Web of Science database without any restrictions on the publication year. The search terms used were: Prevalence AND [oral (sex OR “sexual behavior” OR “sexual practices”)] OR cunnilingus, “oral vaginal contact” OR fellatio OR “oral penile contact”. Studies included in the rapid review were cross-sectional, cohort, and systematic reviews. Case-control, randomized controlled trials, quasi-experimental studies, policy analyses, opinion pieces, and editorials were excluded. The references of the included studies were also studied for additional publications.

We identified 13 studies that met the eligibility criteria (23–35). The summary of the publications is highlighted in Table 1. The practice of oral sex varies widely across populations and contexts. Among young adults in the U.S., 66% of women and 65% of men aged 15–24 reported having engaged in oral sex (28). Prevalence among adolescents globally ranges from 1.7% to 26.6%, with university students reporting higher rates (5% to 46.4%) (31). In Kinshasa, 59% of sexually active individuals reported oral sex, in Singapore, prevalence among female sex workers rose from 27.1% to 81.1% between 1992 and 1997 (23, 32), while in Nigeria, 69% of men who have sex with men and transgender women reported oral sex (34).

**Table 1:** Summary of the publications on the prevalence of oral sex.

Name of author and year of publication	Study objective	Country of study	Population	Sample size	Finding
Wong et al. 2000 (24)	To determine the prevalence of factors associated with consistent condom use during oral sex.	Singapore	Female sex workers	225	The prevalence of oral sex increased from 27.1% in 1992 to 81.1% in 1997, with a concomitant increase in pharyngeal gonorrhea among female sex workers in Singapore. Also, 56.9% consistently used condoms for oral sex compared to 97% for vaginal sex. Significantly higher rates of condom use were found among high-class sex workers and those with negotiation skills.
Malacad and Hess 2010 (25)	To study the oral sex practices of young women in Canada and to explore the attitudes and emotions that young women associate with oral sex	Canada	Women aged 18−25 years	181	Oral sex is as common as vaginal intercourse among young women, with a mean initiation age of 17 for both. Most recent sexual experiences occurred in committed relationships and were associated with positive emotions, though younger women and those not in love with their partner reported more negative emotions.
De Rosa et al. 2010 (26)		USA	Sixth-, seventh- and eighth-grade students at 14 urban public schools in Southern California	4,557	8% had had oral sex. Three percent reported having had oral sex only, and 5% reported having had oral sex and intercourse. Among those who reported intercourse, 69% had used a condom during the last intercourse, and 43% had had multiple partners. Being male, being black, and having at least one friend who had ever been involved in a pregnancy were positively associated with having had intercourse only and both intercourse and oral sex. Intercourse and oral sex were highly correlated.
Dake et al. 2011 (27)	To examine the prevalence of oral sexual activity in rural Midwestern adolescents and the correlates of a series of risk behaviors with oral sexual activity.	USA	Rural middle and high school students in grades 6–12 across 5 rural counties in a Midwestern state	2,000	Slightly more than one-fourth of the students (29%) had engaged in oral sex (9% of middle school and 44% of high school students). Risk behaviors statistically significantly associated with oral sexual behavior were: ever having had sexual intercourse (16.6 times more likely to engage in oral sex), having drunk alcohol in the past 30 days (2.2 times more likely), and having smoked 1 or more cigarettes in the past 30 days (2.0 times more likely).
Brown et al. 2011 (28)	To determine the prevalence of HPV genotypes in the oral cavity and cervix in Peruvian FSWs and examine the association of oral HPV with oral sex practices	Peru	Female sex workers, 18–26 years of age	185	182 participants reported having had oral sex; 95% reported condom use during oral sex with clients and 9.5% with partners. Women who had oral sex more than three times with their partners in the past month were more likely to have oral HPV than women who had oral sex three times or less.
Copen et al. 2012 (29)	To present data on the prevalence of oral sex with opposite-sex partners and the timing of first oral sex relative to first vaginal intercourse among females and males aged 15–24 based on the National Survey of Family Growth (NSFG) data from 2007 to 2010.	USA	15–24 years old	3,242: women 3,104: men	66% of females and 65% of males had ever had oral sex in 2007–2010. Among females, 26% had first oral sex before first vaginal intercourse; 27% had oral sex after intercourse; 7.4% had oral sex on the same occasion as first intercourse; and 5.1% had oral sex, but no vaginal intercourse. Among males, 24% had first oral sex before first intercourse; 24% had oral sex after first intercourse; 12% had oral sex on the same occasion as first intercourse; and 6.5% had oral sex, but no vaginal intercourse.
Cherie and Berhane 2012 (30)	To describe oral and anal sex practices and identify associated factors among high school youth	Ethiopia	High school youth in Addis Ababa	3,840	The overall proportion of people who reported ever having oral sex was 5.4% (190). Of these, 51.6% (98) had oral sex in the past 12 months. Multiple partnerships were reported by 61.2% of the respondents who had oral sex. Reasons for oral and anal sex included prevention of pregnancy, preserving virginity, and reduction of HIV and STIs transmission. Oral sex practice was strongly and significantly associated with the perception of best friends’ engagement in oral sex and having illiterate mothers.
Ma et al. 2013 (31)	To determine the prevalence and correlates of heterosexual oral sex in STD clinic attendees to understand the epidemiology and risks of this type of sexual behaviour	China	Heterosexual attendees who visit sexually transmitted disease	872	6.9% engaged in oral sex over their lifetimes. Of the oral-sex group, 96.6% also engaged in vaginal sex. The correlates for oral sex over a lifetime were high income, high (HIV)-related knowledge, early sex initiation, multiple sexual partners, and being sexually active in the previous 6 months.
Morhason-Bello et al. 2019 (32)	To describe the prevalence of, and motivations for, oral and anal sex among adolescents and adults reporting heterosexual sex in sub-Saharan Africa	Sub-Saharan Africa -	Adolescents and adults reporting heterosexual sex	The majority of the studies (90/103) had participants aged 10 to 49 years	Prevalence of reporting ever practicing oral sex among adolescents, university students, and a combined population of adolescents/adults ranged from 1.7%–26.6%, 5.0%–46.4%, and 3.0%–47.2%, respectively. Higher prevalences of ever practicing oral sex were recorded after 2000 compared to before 2000. Studies conducted among university students reported a relatively higher prevalence of oral sex compared with other groups within the general population.
Carlos et al. 2019 (33)	To describe the prevalence of oral and anal sex among participants attending Voluntary Counseling and Testing in Kinshasa reporting heterosexual sex; and the socio-demographics, perceptions and behavioral factors associated with these practices.	Kinshasa, the Democratic Republic of Congo	15–59-year-old HIV Voluntary Counseling and Testing attendees	797	Among 718 sexually active participants reporting heterosexual sex, 59% had had oral sex, and 18% had oral and anal sex. Among participants reporting “not” having had sex, 6% reported oral sex, and 1% oral and anal sex. Oral sex was linked to daily Internet/mobile phone use, low perceived community HIV risk, risky sexual behaviors (e.g., multiple partners, inconsistent condom use), and pregnancy. Oral sex was less common among married-monogamous individuals. Despite its association with HIV/STIs, oral sex is often not viewed as risky.
Wood et al. 2019 (34)	To determine the prevalence of oral sex practice and tobacco use in a South African patient population	South Africa	18–45 years old	514	115 (2.8%) reported to practice oral sex, most common among the self-identified white participants (41.9%); and among tobacco users than among non-tobacco users (30.9% vs. 20.5%; *p* = 0.022). The practice of OS was more likely among those 18–35 years, but had no significant association with tobacco use.
Robbin et al. 2020 (35)	Estimated the prevalence and factors associated with oral sex practices and characterized oropharyngeal STIs among a cohort of MSM and TGW in Nigeria	Nigeria	Men who have sex with men and transgender women: 22–29	1342	69% reported oral sex practices. Factors associated with increased odds of engaging in oral sex included living with HIV, self-identifying as a woman, mobile phone ownership, receptive anal sex, and multiple male sexual partners. Oropharyngeal STI prevalence was 7% (52/752) and higher among those who engaged in oral sex compared to those who did not.
Kerwin et al. 2014 (36)	The extent of, and factors related to, the knowledge and practice of oral sex in that country	Malawi	Malawian men	1,787 - urban 1,228 – rural	2% of rural and 12% of urban residents had ever received oral sex

Variations also exist across demographics, with 41.9% of self-identified white individuals in South Africa reporting oral sex compared to 2.8% overall (33), 5.4% of Ethiopian high school youth associating the practice with peer norms and maternal education (33), and urban populations in Malawi (12%) reported higher prevalence compared to rural counterparts (2%) (36).

Oral sex is often linked in the literature to risky behaviors, including multiple sexual partners, early initiation, and substance use (26, 30). The inconsistent use of condoms during oral sex was a recurring theme, as seen in Singapore and Peru (23, 27), although oral sex is far less risky than penetrative sex. This inconsistency contrasts with higher condom use rates for vaginal sex (23). Sociocultural factors, such as peer influence and education, significantly shape practices and attitudes (29). Despite its association with sexually transmitted infections like human papilloma virus and gonorrhea (27, 34), it is frequently perceived as low risk and a safer alternative to vaginal intercourse (29, 32).

Oral sex is practiced by adults of all genders, ages, and races (36). Among older adults born after 1942, 80% of men and 70% of women report giving or receiving oral sex (21, 22). The proportion of people who engage in oral sex has increased over the years (21), with younger individuals more likely to report engaging in oral sex (24, 33), and initiation often occurring during adolescence or early adulthood (30). Oral health is linked to intimacy, identity, and well-being. These findings underscore the complexity of addressing oral sex practices in public health strategies. Recognizing the sexual dimensions of oral health can reduce stigma, particularly for marginalized groups like sexual and gender minority individuals and sex workers, and foster more comprehensive public health strategies built on the importance of sexual health for the attainment of oral health.

One of the fundamental challenges with the exclusion of oral sex from discussions about oral health is the associated multiple risks, both in terms of health outcomes and societal perceptions. The risks of this exclusion are compounded by the fact that oral sex has become increasingly common and socially accepted as part of sexual expression (37). Failing to incorporate this into health frameworks can result in misinformed or under-informed public health messaging, stigmatization, and neglect in oral health care. It is associated with the transmission of sexually transmitted infections although this risk is lower than that of vaginal or anal sex (38). In addition, oral cancer (linked to HPV) can be associated with oral sex (39). The absence of discussions about oral sex during oral health education can lead to a lack of preventive measures, such as appropriate vaccinations (e.g., the HPV vaccine), and consideration of barriers like dental dams or condoms during oral sex should an infection be present (40). In addition, people who engage in oral sex may feel a sense of shame or guilt if they perceive their behavior as being unmentionable in healthcare contexts. This may impact their willingness to seek advice or treatment, particularly if they are experiencing oral health issues such as sores, lesions, or other symptoms that could be linked to an orally transmitted sexual infection.

The current definitions of oral health contribute to systemic erasure—the institutional exclusion of marginalized experiences that do not conform to biomedical norms (41). This omission has significant consequences. It pathologizes experiences, renders invisible cultural contexts such as Indigenous oral rituals or African dental modifications, and ignores oral sexual health. The consequences of this erasure are far-reaching, resulting in gaps in public health messaging, limited research on oral health's intersection with sexuality and cultural practices, and policy frameworks that prioritize disease-free metrics over holistic well-being. Systemic erasure in oral health definitions is not a passive oversight—it is a structural barrier to equity. By equating health with biological ‘normalcy, current frameworks exclude the very populations most vulnerable to oral health disparities. Redefining oral health requires dismantling these hierarchies to ensure that no individual's well-being is invalidated by the narrowness of existing definitions.

To avoid the risks associated with systemic erasure, it is essential to frame oral health more inclusively and comprehensively—one that recognizes the full range of behaviors that contribute to both physical and psychosocial well-being. A more holistic definition would address oral health in the context of sexual health, acknowledging that oral sex is a common and significant practice for many individuals. By doing so, we can ensure that individuals receive comprehensive health education that encompasses not only the prevention of disease but also the acknowledgment of various sexual practices and their impact on health.

### A more inclusive definition of oral health and the policy implications

A more inclusive definition of oral health would allow for more open dialogue and reduce the stigma surrounding sexual health discussions. A more inclusive definition of oral health could be:

Oral health is a state of well-being that includes the absence of disease in the oral cavity, its ability to function in ways that allow individuals to speak, eat, smile, taste, touch, chew, swallow, and convey a range of emotions through facial expressions comfortably with consideration of the psychological, social, and sexual factors that influence oral well-being.

This definition incorporates both the traditional aspects of oral health (e.g., the physical condition of the mouth and teeth) and the modern realities of sexual health, recognizing the need for preventive education and care. By including sexual factors (i.e., oral sex) in the discussion of oral health, healthcare systems can better address the full spectrum of behaviors that influence health outcomes and provide individuals with the tools they need to protect themselves and maintain their overall well-being.

Oral sex is a widespread practice across diverse populations, spanning countries, races, ages, and genders. However, this paper moves beyond prevalence statistics to argue that current oral health definitions fail to recognize minority experiences as valid aspects of well-being. The paradox is clear: while oral sex is practiced globally, its omission from oral health definitions reflects systemic biases that privilege particular norms. Our proposed inclusive definition integrates psychosocial and sexual dimensions, challenging dominant hierarchies that position minority experiences as peripheral rather than integral to oral health. Silence perpetuates disparities. Expanding definitions to reflect lived realities is essential in dismantling stigma and ensuring equitable care.

Moving from evidence to action requires structural change. Medical education must equip oral health providers to discuss oral sexual health without judgment, addressing gaps in promoting oral health that is not based exclusively on the experience of the majority. Public health campaigns should adopt inclusive messaging, recognizing the influence of education on sexual health behaviours. In addition, research funding must prioritize marginalized voices, ensuring that oral health frameworks reflect the realities of diverse communities. Without such shifts, the biopsychosocial model remains theoretical, failing those whose well-being is shaped by more than biomedical factors alone. By prioritizing inclusivity, oral health can move from a tool of exclusion to one of empowerment, ensuring no individual's well-being is rendered invisible by narrow definitions.

A truly inclusive oral health policy acknowledges and respects the diversity of individual experiences and identities. Expanding current definitions of oral health to encompass aspects of consensual sexual pleasure, psychosocial well-being, and cultural nuances would allow health systems to foster environments where all individuals feel respected. By doing so, policy frameworks can better address the needs of all people who engage in oral sexual practices, including minority populations.

One critical step toward inclusivity is re-evaluating educational content for health professionals. Incorporating culturally competent approaches in the dental and public health educational curriculum, especially regarding oral sexual health, can reduce stigma and promote discussions on oral health in the context of intimacy and pleasure, which would support this shift. These educational advancements would not only increase provider competency but also empower healthcare workers to engage respectfully and openly with patients, regardless of their backgrounds or sexual practices. By normalizing discussions around oral sexual health, providers can ensure that patients are comfortable discussing their full health histories, which is essential for comprehensive care. This shift in policy would require collaborative efforts among dental associations, policymakers, and healthcare professionals.

## Conclusion

The current definitions of oral health by the FDI and WHO, while foundational, are fundamentally limited by their narrow focus on pain-free functionality. These frameworks fail to acknowledge a critical truth: oral health is a dynamic interplay of biological, psychological, and sociocultural factors—including practices like oral sex that hold diverse meanings across populations and communities. By equating health solely with the absence of disease, these definitions invalidate the lived realities of individuals for whom oral health encompasses pleasure, intimacy, or culturally specific practices. This exclusion is not merely an oversight; it is a form of systemic erasure that perpetuates stigma, particularly for sexual and gender minorities, older adults, and marginalized populations whose sexual practices and health needs are rendered invisible.

To truly advance equity in oral health, we must reject definitions that prioritize biomedical norms over human diversity. An inclusive framework must recognize that well-being is not universally defined by comfort—for many, it involves consensual practices that may challenge traditional health paradigms. Such a shift would transform clinical practice: dismantling taboos, empowering providers to deliver culturally competent care, and validating patients who have long been marginalized by rigid health standards. Policymakers must act urgently to expand these definitions, ensuring they reflect the full spectrum of human experience. The evidence is clear: when practices vary so widely across ages, cultures, and identities, our definitions must be equally expansive. Anything less perpetuates harm under the guise of neutrality.

## Data Availability

Publicly available datasets were analyzed in this study. This data can be found here.
